# Transcriptional regulators of *Arabidopsis* secondary cell wall formation: tools to re-program and improve cell wall traits

**DOI:** 10.3389/fpls.2014.00192

**Published:** 2014-05-14

**Authors:** Rakesh Bhatia, Maurice Bosch

**Affiliations:** Institute of Biological, Environmental and Rural Sciences, Aberystwyth UniversityAberystwyth, UK

**Keywords:** secondary cell wall, transcription factors, *Arabidopsis*, lignin, recalcitrance, lignocellulosic biomass, genetic engineering, biofuels

Secondary cell walls (SCWs) comprise the main portion of plant biomass and represent an abundant renewable resource of polysaccharides for conversion into sugars and subsequent fermentation to liquid biofuels. An upswing in lignocellulosic biomass-derived biofuels, including from grass and woody energy crops and agricultural residues, offers significant environmental and socio-economic benefits over the use of dwindling and polluting non-renewable fossil fuels. A key challenge for realizing commercial biofuel production resides in the economically unfeasible sugar yields after enzymatic hydrolysis of lignocellulosic biomass. This low saccharification efficiency is caused by the recalcitrant nature of the cell wall owing to the crystallinity of cellulose and interactions between the polysaccharides and lignin polymers within the cell wall matrix (Himmel et al., 2007; Demartini et al., 2013). Overcoming biomass recalcitrance has generally been tackled with costly and energy intensive pre-treatments, which so far have met bioconversion requirements with limited success. One alternative path to optimize the digestibility of lignocellulose for sustainable biofuel production is the genetic manipulation of SCW characteristics via transcription factors (TFs), powerful tools to orchestrate the biosynthesis and deposition of the main SCW components cellulose, lignin, and hemicellulose.

Research efforts have revealed an extensive, complex and hierarchical regulatory network of SCW-related TFs predominantly comprising NAC and MYB family members in *Arabidopsis* (Handakumbura and Hazen, 2012; Hussey et al., 2013). Delineating the topology and dynamics of this regulatory network will require exploring its nodes, hubs, and edges. Cassan-Wang et al. (2013) confront these fundamental goals by tallying new TFs to the network regulating SCW formation in *Arabidopsis*. Their innovative strategy to identify transcriptional regulators involved the exploitation of several *Arabidopsis* SCW-related transcriptome datasets combined with *in silico* expression and histology-based phenotyping screens. Since lignin is commonly acknowledged to inhibit biomass digestibility, their systematic approach proved useful as six T-DNA lines from candidate TFs revealed altered lignin deposition profiles; including hyper- and hypo-lignification as well as ectopic lignin deposition (Cassan-Wang et al., 2013). Co-expression analysis suggests that some of these TFs not only play a role in lignin biosynthesis, but also regulate other parts of the SCW formation program including cellulose and xylan biosynthesis. It is noteworthy that the majority of T-DNA/RNAi lines corresponding to candidate SCW-related TFs did not reveal a lignin phenotype. The fact that their candidate list of TFs contains known SCW regulators validates the robustness of their identification strategy. However, it is well established that redundancy exists among TFs and the authors, therefore, rightly touch upon the need for analyzing over-expressor lines and/or multiple gene knock-out mutants of homologous TFs in order to increase the likelihood of a detectable SCW phenotype and understanding of gene function. One approach that has been particularly successful in addressing this problem of functional redundancy is that of dominant repression (Hiratsu et al., 2003). The expression of chimeric repressors (specific TF fused to a repression domain) induces loss-of-function phenotypes in transgenic plants by dominating the activity of both endogenous and functionally redundant TFs, facilitating the analysis of TF function in SCW formation (Zhong et al., 2007; McCarthy et al., 2009). The identification of the lignin mutants by Cassan-Wang et al. (2013) was ultimately based on histological observations and more extensive compositional analysis might increase the number of SCW-related mutants beyond the six already identified. For instance, it is known that certain SCW-related TFs regulate specific lignin monomers (Zhao et al., 2010; Öhman et al., 2013). The ratios of these monomers may therefore have been shifted in some TF mutants, without causing an obvious phenotype for plant growth or lignin staining but potentially affecting recalcitrance and saccharification yield.

The list of potential SCW-related TFs identified by Cassan-Wang et al. (2013) highlights the expanding complexity of SCW regulation, including involvement of TF families for which participation in SCW regulation is not yet well established. This encompasses members from the AP2/ERF and Aux/IAA TF-families, also identified in other screens targeted at genes involved in SCW formation (Bosch et al., 2011; Hirano et al., 2013). Involvement of these families in the regulatory SCW network suggests the participation of complex ethylene- and auxin-signaling pathways. In particular AP2/ERF TFs represent attractive targets for genetic engineering to enhance crop performance in terms of stress tolerance or higher yield as well as SCW properties for bioconversion purposes (Ambavaram et al., 2011; Vahala et al., 2013).

Although TFs provide attractive targets for altering complex cell wall traits, the intricacy of regulatory networks and their integration with different metabolic and signaling pathways, means that changing the levels of a certain TF can result in pleiotropic effects. In accordance, Cassan-Wang et al. (2013) observed delayed flowering in their hyperlignified *hb5* line and earlier flowering in the hypolignified *blh6* and *zinc finger* lines. This corroborates previous reports that some TFs of the SCW thickening and xylem formation program are coupled with the flowering induction program (Melzer et al., 2008; Sibout et al., 2008; Xu et al., 2012), exemplifying the danger of unintended consequences when altering SCW synthesis due to its tight integration with other developmental processes.

As eudicots and monocot grasses exhibit differences in cell wall composition and architecture as well as morphological and anatomical distinctions (Vogel, 2008; Sarkar et al., 2009), one could argue that they may have evolved a unique transcriptional regulatory network controlling SCW biosynthesis. Interestingly, phylogenetic and co-expression analysis illustrate evolutionary conservation of the transcriptional regulators in SCW biosynthesis (Ruprecht et al., 2011; Zhong et al., 2011). Discovering SCW-related TFs in the model system *Arabidopsis* should therefore provide compatible tools for the engineering of woody and grass energy crops alike. Indeed, researchers developing switchgrass as a dedicated bioenergy feedstock have exploited this apparent conservation; over-expression of the MYB TF *PvMYB4*, an orthologue of *Arabidopsis AtMYB4*, results in repression of genes involved in lignin biosynthesis, leading to reduced cell wall recalcitrance and a more than doubling of the bioethanol yield (Shen et al., 2013). This and other examples clearly indicate the potential of translating results from *Arabidopsis* to tailor the biochemistry of SCWs in lignocellulosic feedstocks for improved performance. An important consideration is the trade-off between reduced cell wall recalcitrance and biomass yield, often encountered in genetic engineering efforts aimed at modifying SCWs. New synthetic biology approaches may alleviate such growth penalties. This is illustrated by a study in which SCW biosynthesis and TF genes were used to re-program the spatial deposition patterns of lignin and polysaccharides in *Arabidopsis*, doubling the sugar yields after mild pre-treatment and enzymatic hydrolysis without affecting plant growth (Yang et al., 2012).

Knowledge of the TFs involved in SCW formation can also be exploited for breeding of cultivars with improved cell wall characteristics. Several QTLs for lignin- and saccharification-related traits in maize and sorghum have been shown to co-localize with TFs that regulate SCW biosynthesis (Barriere et al., 2012; Wang et al., 2013), providing inroads for improving desired SCW traits related to forage feeding value and saccharification yield through marker-assisted breeding.

In summary, Cassan-Wang et al. (2013) have highlighted an effective approach for identifying novel TFs involved in *Arabidopsis* SCW formation, providing a solid platform for more detailed functional analysis. The emerging picture is that of an increasingly complex regulatory network underlying SCW formation. Fitting together the pieces of the transcriptional puzzle will be challenging but the process will provide us with essential knowledge and tools to re-program and improve cell wall related traits.

## Conflict of interest statement

The authors declare that the research was conducted in the absence of any commercial or financial relationships that could be construed as a potential conflict of interest.
